# Plague from Eating Raw Camel Liver

**DOI:** 10.3201/eid1109.050081

**Published:** 2005-09

**Authors:** Abdulaziz A. Bin Saeed, Nasser A. Al-Hamdan, Robert E. Fontaine

**Affiliations:** * King Saud University College of Medicine, Riyadh, Saudi Arabia;; † Ministry of Health, Riyadh, Saudi Arabia;; ‡ King Faisal Specialist Hospital and Research Centre, Riyadh, Saudi Arabia;; § Centers for Disease Control and Prevention, Atlanta, Georgia, USA

**Keywords:** plague, Yersinia pestis, pharyngitis, gastroenteritis, foodborne, meningitis, camels, gerbillinae, Meriones libycus, dispatch

## Abstract

We investigated a cluster of 5 plague cases; the patients included 4 with severe pharyngitis and submandibular lymphadenitis. These 4 case-patients had eaten raw camel liver. *Yersinia pestis* was isolated from bone marrow of the camel and from jirds (*Meriones libycus*) and fleas (*Xenopsylla cheopis*) captured at the camel corral.

Human plague is acquired most often from the bites of infected fleas that leave their rodent hosts. Sporadic plague has also been attributed to domestic dogs and cats that may transport either *Yersinia pestis* in their mouths or infected fleas from rodent hosts to humans (*1*). Bubonic, pneumonic, or pharyngeal plague may develop in domestic cats and infect humans directly (*2*). However, humans rarely become infected when handling and preparing the carcasses of wild animals (*3*). Although domestic cats and other carnivores may be infected by eating infected animals, only 1 previous report raises the possibility of human plague infection from eating meat of an infected animal (*4*).

## The Study

In February 1994, we investigated a cluster of 5 plague cases in Goriat, a town of 50,000 persons in a remote desert area in northwestern Saudi Arabia. On February 18, a 26 year-old-woman was admitted to the provincial hospital for severe pharyngitis and tonsillitis. Given the striking swelling of her neck, local clinicians suspected diphtheria. Since 2 of the patient's relatives had also been hospitalized in the previous 2 days with similar illnesses, the hospital called for assistance from the local preventive medicine specialist. He had seen similar cases in 1984 and suspected pharyngeal plague.

Through interviews with physicians and review of hospital admissions, we identified 5 patients, including the index case, who had been hospitalized with suspected plague or plague pharyngitis. The patients included a 9-year-old girl and 4 adults (2 men and 2 women, age range 18–35 years). Symptoms developed in 1 patient on February 15 and in 4 patients on February 16. All had fever (39°C–40°C), chills, malaise, myalgias, vomiting, headache, and delirium. Leukocyte counts ranged from 11,000 to 88,000/mL. Chest radiographs were normal in all 5 patients. Four had severe pharyngitis; 3 of them had dysphagia, tender submandibular lymphadenitis, and tonsillar enlargement. The fourth patient, the 9-year-old girl, had severe abdominal pain and tenderness on abdominal palpation, profound hypotension (blood pressure 60/30 mm Hg), and a generalized hemorrhagic rash. This patient and the 26-year-old index patient (blood pressure 90/60 mm Hg) died. These 4 patients with pharyngitis did not have buboes or lymphadenitis at any other site. The patient without pharyngitis had axillary lymphadenitis and cellulitis of his right arm; he had cut his arm while killing a sick camel on February 13. None of the patients had skin lesions that suggested recent flea bites.

*Y. pestis* was isolated from the blood of the patient with pharyngitis who died and from the spinal fluid of the patient with abdominal pain. Identification was confirmed by phage lysis and direct fluorescent antibody staining. Indirect hemagglutination for plague was positive in convalescent-phase sera from the 3 survivors from whom *Y. pestis* was not isolated.

The patients were from 4 related families, 2 from Goriat and 2 from a village 20 km from this town. The adult family members denied seeing rodents around their homes or being bitten by fleas or other biting insects. All families owned camels. The male head of each family traveled to the desert daily to allow his camels to graze. These men reported that several of their camels had recently died. We observed 3 camel carcasses in the desert near a corral where the camels were fed grain and hay to supplement their grazing.

The meat from the sick camel that had been butchered on February 13 was shared among 11 families (106 members). No other food was shared among these families. The 4 patients with pharyngeal plague were among 37 people who had eaten this camel meat; 1 patient with bubonic plague (the man who slaughtered the camel) was among the 69 people who had not eaten the meat (risk ratio [RR] 7.7, p<0.05, Fisher exact test). Moreover, pharyngeal plague developed in 4 of 6 patients who had eaten raw camel liver, but not in 31 persons who had eaten only cooked camel meat or liver (RR not defined, p<0.01, Fisher exact test).

We isolated *Y. pestis* from a sample of leftover camel meat containing bone and marrow. Jirds (*Meriones libycus*), jird carcasses, rodent burrows, and rodent excreta were found at the camel corral. *Y. pestis* was isolated from the blood and liver of live jirds collected from the camel corral and from fleas (*Xenopsylla cheopis*) combed from these jirds.

## Conclusions

This investigation confirms that human plague with pharyngeal and gastrointestinal symptoms can result from eating infected raw camel liver. Only 1 published report has proposed this method of infection. In 1976, in a small, remote Libyan village, 13 plague cases occurred after a sick camel was slaughtered and its meat eaten (*4*). However, as a source of infection eating camel meat could not be distinguished from droplet transmission, percutaneous exposure during camel killing or handling fresh meat, or flea bites. Moreover, plague infection was not found in the camel, and the human pharyngeal plague might have resulted from crushing fleas between the teeth while grooming (*5*).

The 4 patients with pharyngeal plague had symptoms similar to those of domestic cats with plague in New Mexico, where 46% of plague-infected cats had submandibular lymphadenitis (*2*). These feline cases were thought to result from eating infected prey. Similarly, since human plague patients had submandibular lymphadenitis, tonsillitis, pharyngitis and dysphagia, or severe gastrointestinal symptoms, this suggests ingestion as the route of exposure.

Christie et al. suggested that among domestic animals, camels may be an important plague host because their wide-ranging behavior increases the chance of coming into contact with natural plague foci (*4*). Our investigation indicates another scenario, with evidence of a plague epizootic at a fixed site where camels were corralled. Russian investigators have proposed several methods of natural infection of camels: bites from rodent fleas, mechanical transmission from ticks, eating feed contaminated with excreta of infected rodents, and eating dead rodents along with their feed (*6*). All of these possibilities existed in this outbreak.

This unusual profile of human plague was recognized because several related patients with life-threatening illnesses were seen at a hospital over a 2-day period and a medical officer recognized the illness. Sporadic cases of pharyngeal or gastrointestinal plague would be less likely to attract the attention of medical or public health workers. Clinicians and public health officers, particularly in the Middle East, North Africa, and Central Asia, should be alert for sporadic cases of pharyngeal or gastrointestinal plague so that curative and preventive measures can be promptly initiated.
